# Minimally processed foods have a higher total antioxidant content compared to processed and ultra-processed foods: results from an analysis of 1946 food items

**DOI:** 10.1017/S0007114524002800

**Published:** 2024-12-28

**Authors:** Anthony J. Basile, Anaissa Ruiz-Tejada, Alex E. Mohr, Steven Stanley, Ellinor Hjelm, Karen L. Sweazea

**Affiliations:** 1 School of Life Sciences, Arizona State University, 427 E. Tyler Mall, Tempe, AZ 85281, USA; 2 Department of Human Ecology, State University of New York at Oneonta, 108 Ravine Parkway, Oneonta, NY 13820, USA; 3 College of Health Solutions, Arizona State University, 500 N 3rd St, Phoenix, AZ 85004, USA

**Keywords:** Total Antioxidant Content, NOVA Classification, Food Processing, Ultra-processed Foods

## Abstract

Ultra-processed foods (UPF), per the NOVA Classification, provide a major source of calories within modern food systems and are associated with poor health outcomes related to chronic inflammation. Dietary antioxidants play a key role in preventing disease; however, the relationship between the NOVA Classification and the total antioxidant content (TAC) of foods is not well characterised. We hypothesised that TAC would be highest in minimally processed food (MPF), lower in processed food (PRF) and lowest in UPF. TAC data for 3137 animal-based, mixed and plant-based food items were obtained from a published dataset. After data cleaning, 1946 food items and their TAC values were analysed using two hierarchal linear models (alpha: *P* < 0·05). MPF had the highest mean TAC (10·79 (sem 0·87) mmol/100 g) and were 11·31-fold and 10·72-fold higher than PRF and UPF, respectively (*P* = 0·023). Plant-based and mixed foods had a higher mean TAC (8·55 (sem 0·68) and 1·12 (sem 0·11) mmol/100 g, respectively) and were 22·67-fold and 2·98-fold higher compared with animal-based foods (*P* < 0·001). Food processing did not change mean TAC in mixed and animal-based foods; however, plant-based MPF had a higher mean TAC (11·49 (sem 0·93) mmol/100 g) and were 9·88-fold and 15·12-fold higher compared with plant-based PRF and UPF, respectively (*P* < 0·001). Mean TAC differed between NOVA processing groups for three categories of food: vegetables, beverages and beans, nuts and seeds (*P* < 0·001). Across all food items, and especially plant-based foods, mean TAC decreased with food processing. The lower TAC of UPF may at least partially explain why their consumption promotes inflammatory chronic disease.

Ultra-processed foods (UPF) make up more than 50 % of the diets of adults and children in the UK and the USA^(1–3)^, and high consumption of UPF is also seen in other countries^(3–5)^. Consumption of UPF is positively associated with numerous conditions involving chronic inflammation such as type 2 diabetes^(6)^, depression^(7)^, obesity^(8)^, colorectal cancer^(9)^ and all-cause mortality^(10,11)^. In addition, these foods are readily available and cost less per calorie^(12)^. The commonly used NOVA Classification^(12–14)^, first developed in 2010^(15)^, categorises foods by their degree of processing into four main groups: unprocessed and minimally processed foods (MPF), processed culinary ingredients (CPF), processed foods (PRF) and UPF^(16)^.

MPF includes unprocessed foods obtained from nature without the addition of culinary ingredients^(17)^. As the name implies, this group allows for several techniques that minimally process foods such as crushing and grinding, filtering, boiling, roasting, pasteurisation, refrigeration and freezing, packaging, as well as fermentation without the use of alcohol. Examples of foods within this NOVA group include grains, flours, beans, nuts, fruits, vegetables, fungi, seafood, meat, poultry, eggs, milk and pure juices^(17)^. CPF includes items obtained from MPF that are intended for use in cooking and food preparation to increase the palatability of foods or to aid in the cooking process such as sea salt, sugar, molasses, honey, olive or seed oils, butter, lard and plant-based starches^(17)^. When CPF are added to MPF, the resulting food product becomes PRF^(17)^. In addition to enhancing flavour, this type of processing enables the preservation of foods such as fermentation without the use of alcohol, canning, bottling and the addition of antioxidants or antimicrobial preservatives^(17)^. Examples of PRF include beer and wine, cheese, breads, canned or bottled vegetables and fruits, sweetened or salted seeds and nuts, as well as smoked or salted canned meats and fish^(17)^. UPF are often ready to eat and contain additives other than common cooking ingredients (e.g. salt, sugar, oils and fats), such as flavours, colours, artificial sweeteners, emulsifiers, bulking or anti-caking agents and a multitude of other additives^(16–18)^. These ingredients are used to increase the palatability of unprocessed or MPF and their culinary preparations, in addition to preserving foods and extending their shelf-life^(17,18)^. Examples of UPF include carbonated sweetened beverages, packaged pastries and other baked goods, drink mixes, ready-made sauces, fortified products, hot dogs, instant soups and meals, energy drinks and infant formula^(17)^. Overall, UPF contribute to deleterious metabolic effects and inflammation and thus represent a serious challenge for public health^(19)^.

Dietary antioxidants play a key role in promoting oxidative balance by providing a hydrogen atom to quench reactive oxygen species and stop oxidative chain reactions^(20)^. Oxidative stress occurs when there is an imbalance between antioxidants and reactive oxygen species, which can alter the structure and function of proteins and lead to a state of inflammation that may contribute to numerous chronic diseases (e.g. CVD, diabetes and cancer)^(21)^. Thus, consuming foods high in antioxidants may be a good strategy to prevent oxidative stress-related diseases^(22)^ and mortality^(23)^. Major sources of dietary antioxidants include tea, dietary supplements, fruit and fruit juices^(24)^. Generally, plant-based foods are rich in antioxidants (e.g. fruits and vegetables)^(25)^; however, processed fruits are associated with a lower antioxidant content and an overall degradation in the nutritive quality^(26)^. Moreover, various cooking methods, such as steaming^(27)^ and roasting^(28,29)^, can decrease total antioxidant content (TAC) of foods. These cooking methods fall under the MPF and PRF of the NOVA Classification. Currently, it is unknown how food processing (measured by NOVA Classification), and more specifically ultra-processing, affects TAC of foods classified either by food type (i.e. Animal or Plant-based and Mixed) or specific food categories (i.e. alcohol; beverages; dairy; fish, meat and poultry: FMP; beans, nuts and seeds: BNS; grains; fruit; vegetables; and fats and sweets: F&S).

If food processing results in a lower TAC value for food, this could be one of the mechanisms by which consumption of these foods is associated with inflammatory chronic disease. Therefore, the purpose of this study was to use the TAC of foods, beverages, spices and herbs, from a provided database^(25)^, to examine how mean TAC is altered by NOVA Classification food processing level (MPF, PRF and UPF). To date, an analysis of TAC through the NOVA Classification has yet to be conducted. This study had three aims: (1) to determine how food processing affects TAC for all food items in the database, (2) to determine how food processing affects TAC by food type (i.e. plant based, animal based and mixed foods) and (3) to determine how food processing affects TAC within food categories (alcohol; beverages; dairy; fish, meat, and poultry: FMP; beans, nuts and seeds: BNS; grains; fruit; vegetables and fats and sweets: F&S). It was hypothesised that TAC would be highest in MPF, lower in PRF and lowest in UPF, with a similar trend seen for the food types and food categories.

## Experimental methods

### Study design and total antioxidant data

The present study was a secondary analysis of a published dataset that measured the TAC various foods obtained either by the original investigators from local markets and stores worldwide or the USA Department of Agriculture National Food and Nutrient Analysis Program^(25)^. TAC was measured via the ferric reducing antioxidant power assay of homogenised foods prepared according to package instructions when necessary (mmol/100 g)^(25)^. TAC data for 3137 foods within identified food types (animal based, plant based and mixed) were collected from the published database^(25)^. Non-food items (herbal medicine and vitamin supplements) were removed yielding 2951 food items available for the current analyses.

### Classifying food items by food group and processing

Foods were coded into nine food categories that included the seven USA Department of Agriculture MyPyramid food categories (dairy; fish, meat and poultry: FMP; beans, nuts and seeds: BNS; grains; fruit; vegetables and fats and sweets: F&S)^(12)^ along with beverages and alcohol (note: fruit and vegetable juices were included in fruit and vegetable categories). Mixed foods were assigned to whichever food category supplied the most volume for a given food (e.g. pizza was coded as a grain). Researchers were trained in identifying food processing levels via the NOVA Classification through reviewing literature^(18,19)^ and conducting several rounds of practice food coding. Next, included foods were coded into the four NOVA food processing classification groups: unprocessed and minimally processed (MPF), processed culinary (CPF), processed (PRF) and UPF per Monteiro et al.^(18,19)^. When possible, the Internet was used to obtain ingredient information for food items as necessary (e.g. reviewing product websites, ingredients listed on packaging or recipe ingredients). If ingredient information was not available, coding was based on provided food item details alone. Coding of food items into food categories and processing groups was conducted first independently and then to agreement by four researchers in two pairs to mitigate the risk of incorrectly classifying foods (AJB and SS; ART and EH; each group coded half the food groups). Both coding pairs had high initial agreement on NOVA group classification (92 and 93 %) and food category coding (94 and 87 %). Disagreements were resolved by a third member of the coding team.

### Data cleaning the food item and total antioxidant content dataset

After NOVA coding was complete, the food items were further cleaned to strengthen the analysis. Food items with different varieties or flavours (e.g. apples, salad dressings and chocolates) and preparations (e.g. raw, boiled, microwaved and steamed) were included to capture the wide variety of foods and their associated cooking methods. However, undiluted and unprepared foods requiring preparation were removed from the list (e.g. dried tea, cake mixes and concentrated juices), but raw cooking ingredients that could be consumed alone or that required cooking (e.g. wheat flour, oatmeal and raw meats), along with their cooked forms, were retained for analysis. Lastly, duplicate food items with the same NOVA group, of different brands or sources (e.g. canned beans from different brands), sizes (e.g. large and small raisins) and pluralities (e.g. carrot and carrots) were averaged together (e.g. there were eleven data points for tomato juice, twenty-seven red wines and six chocolate chip cookies). These methods helped ensure the initial dataset did not skew the analysis through duplicated items and the inclusion of inedible food items, particularly those with concentrated antioxidant content (e.g. dried teas). After coding and data cleaning were complete, CPF data were removed as these foods are not directly eaten and instead used for cooking, thus yielding 1946 food items for analysis (see online Supplementary Fig. 1 for data selection flowchart summary).

### Statistical analysis

Normality statistics (Kolmogorov–Smirnov tests and skewness and kurtosis z-scores) and probability plots (Q-Q plots and histograms) were generated to test normality assumptions, and log transformations were performed as appropriate. To determine the effect of level of food processing on TAC, two hierarchal linear models were employed^(30)^. In the first model, NOVA Classification (MPF, PRF and UPF) and food type (plant based, animal based and mixed) were added as main effects with a random intercept for each food item (*n* 1946). Additionally, to assess whether NOVA Classification was different between types of food, an interaction term was added to the model. A second model was constructed to better determine which food categories (alcohol, beverages, BNS, dairy, fruits, F&S, grains, FMP and vegetables) might be more predictive of TAC by NOVA Classification. Here, the main effects were NOVA Classification and food category with their interaction term. In both models, multiple comparisons were made on generated estimated marginal means for main effects and interaction terms with Bonferroni *post hoc* tests. All analyses were performed using Statistical Package for the Social Sciences version 27.0 for Windows (IBM). The *α*-level was set at a significance of *P* < 0·05. Data are displayed as untransformed mean and standard error of the mean values.

## Results

### Aim 1: Total antioxidant content of all individual food items grouped by NOVA Classification

**Figure 1.**
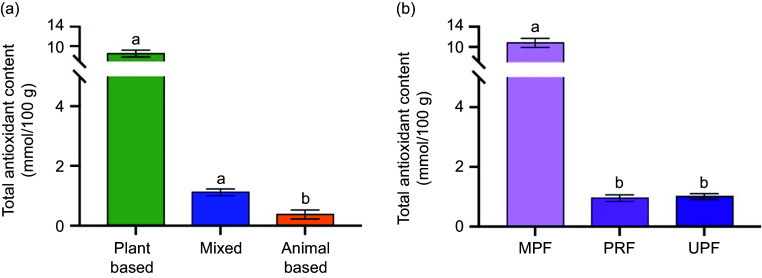
Mean total antioxidant content by food type and processing level for 1946 food items. Plant based and mixed foods had higher total antioxidant content compared with animal-based foods (Panel A; *P* < 0·001). Minimally processed foods had a higher total antioxidant content compared to processed and ultra-processed foods (Panel B; *P* = 0·005). Non-transformed means and standard error of the mean are shown and means with different letters are significantly different from each other. Analysed via hierarchal linear model. MPF, minimally processed foods; PRF, processed foods; UPF, ultra-processed foods.

A total of 1946 food items were analysed that resulted in *n* 652 (34 % of overall food items) UPF, *n* 308 (16 %) PRF and *n* 986 (51 %) MPF. MPF had the highest mean TAC (10·79 (sem 0·87) mmol/100 g) and were 11·31-fold and 10·72-fold higher than PRF and UPF foods, respectively (*P* = 0·023; Fig. 1).

### Aim 2: Total antioxidant content of food types

**Figure 2.**
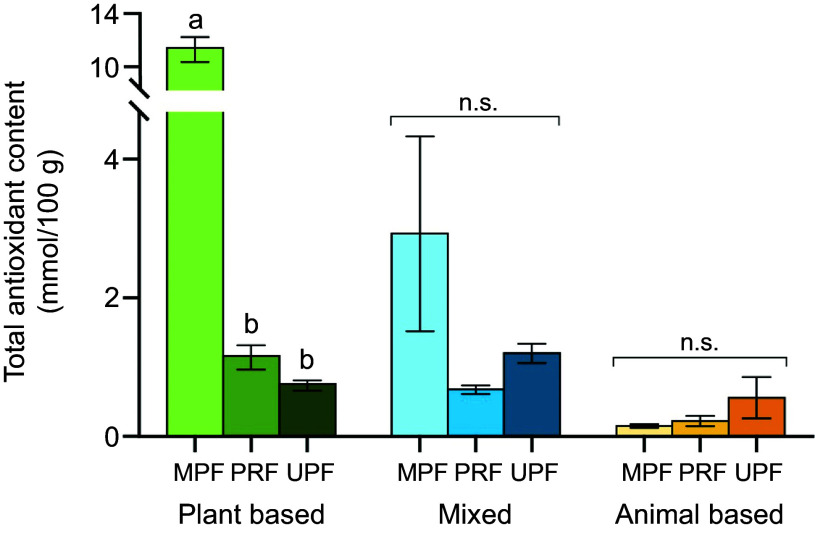
Effect of food processing on mean total antioxidant content within food types for 1946 food items. Minimally processed plant-based food had a higher total antioxidant content compared to processed and ultra-processed plant-based foods (*P* < 0·001). Non-transformed means and standard error of the mean are shown and means with different letters are significantly different from each other. Analysed via hierarchal linear model. MPF, minimally processed foods; PRF, processed foods; UPF, ultra-processed foods; n.s., not significant.

Food items were divided into animal based, plant based or mixed. Plant based and mixed foods had a higher mean TAC (8·55 (sem 0·68) and 1·12 (sem 0·11) mmol/100 g, respectively) and were 22·67-fold and 2·98-fold higher compared with animal-based foods (*P* < 0·001; Fig. 1). Food processing did not change mean TAC in mixed and animal-based foods; however, plant-based MPF had higher mean TAC (11·49 (sem 0·93) mmol/100 g) and were 9·88-fold and 15·12-fold higher compared with plant-based PRF and UPF, respectively (*P* < 0·001; Fig. 2).

### Aim 3: Total antioxidant content of food categories

Individual food items were sorted into the following nine food categories: dairy; FMP; BNS; F&S. Vegetables were the food category with the highest mean TAC (15·27 (sem 1·30) mmol/100 g; Table 1) and were higher than BNS, grains, FMP and dairy (Table 2; *P* < 0·001). Food processing altered the mean TAC for several food categories: Vegetables, BNS and Beverages (*P* < 0·001; Table 1). Of these groups, the greatest fold difference was between MPF vegetables and PRF and UPF Vegetables (31·07-fold and 21·62-fold, respectively; *P* < 0·001).

**Table 1.** Total antioxidant content of food categories and within food group processing levels for 1946 food items (Mean values with their standard errors)

	All food items			Minimally processed			Processed			Ultra-processed		
	*n* and mean (sem) total antioxidant content mmol/100 g											
Food categories	*n*	Mean	sem	*n*	Mean	sem	*n*	Mean	sem	*n*	Mean	sem
Vegetables	605	15·270	1·304	428	21·252	1·764^a^	105	0·684	0·077^b^	72	0·983	0·275^b^
Fruits	252	4·299	1·149	225	4·550	1·280	22	2·558	1·315	5	0·650	0·146
Beans, nuts seeds	154	2·117	0·389	100	2·982	0·581^a^	34	0·724	0·145^b^	20	0·161	0·019^b^
Fats and sweets	190	2·291	0·265	1	n/a		12	2·038	0·323^b^	177	2·258	0·279^b^
Beverages	118	1·584	0·211	70	2·224	0·303^a^	6	3·508	1·179^a^	42	0·242	0·053^b^
Alcohol	72	1·103	0·129	–	–		66	1·184	0·136	6	0·212	0·067
Grains	318	0·526	0·038	105	0·424	0·064	36	0·309	0·0578	177	0·631	0·055
Fish, meat and poultry	159	0·373	0·143	27	0·213	0·053	27	0·236	0·052	105	0·449	0·215
Dairy	78	0·187	0·032	30	0·155	0·067	–	–		48	0·208	0·031

Difference in mean TAC between food categories: *P* < 0·001; means with different letters are significantly different from each other; food categories listed in descending order of TAC for all food items.

**Table 2.** Difference in mean total antioxidant content between food categories for 1946 food items

	VEG	Fruits	BNS	F&S	BEV	Alcohol	Grains	FMP	Dairy
Food categories									
Vegetables	–	–	–	–	–	–	–	–	–
Fruits	n.s.	–	–	–	–	–	–	–	–
Beans, nuts and seeds	< 0·001	n.s.	–	–	–	–	–	–	–
Fats and sweets	n.s.	n.s.	n.s.	–	–	–	–	–	–
Beverages	n.s.	n.s.	n.s.	n.s.	–	–	–	–	–
Alcohol	n.s.	n.s.	n.s.	0·013	n.s.	–	–	–	–
Grains	< 0·001	n.s.	n.s.	0·012	0·011	n.s.	–	–	–
Fish, meat and poultry	< 0·001	0·029	n.s.	0·003	< 0·001	n.s.	n.s.	–	–
Dairy	< 0·001	0·023	n.s.	0·002	< 0·001	n.s.	n.s.	n.s.	–

BNS, beans, nuts and seeds; FMP, fish, poultry and meat; F&S, fats and sweets; VEG, vegetables; BEV, beverages; n.s., not significant.

Difference between mean TAC of food categories: *P* < 0·001; food categories listed in descending order for mean TAC, see Table 1 for data.

## Discussion

The purpose of this study was to better understand how food processing, as defined by the NOVA Classification, affects the TAC of foods. Determining this relationship could help provide a mechanism for the identified relationship between consumption of UPF and negative health outcomes related to oxidative stress and inflammation^(31–34)^. The results of the present study support the hypothesis that food processing affects TAC. Specifically, TAC is lower for any food processing level above minimally processed (i.e. PRF and UPF). In addition, plant based and mixed foods have a higher TAC compared with animal-based foods. Further, an interaction between plant-based foods and food processing revealed that the mean TAC for plant-based MPF was higher than the mean TAC of plant-based PRF and UPF. Similarly, several plant-based MPF food categories (vegetables and BNS) had higher mean TAC compared with PRF or UPF within these food categories. Minimally processed and processed beverages also had higher mean TAC compared with UPF. These results support the hypothesis, but only when comparing MPF to PRF and UPF for all food items, plant-based foods and some food categories (i.e. vegetables, BNS and beverages). The current results align with a previous small diet analysis of patients with severe obesity prior to undergoing gastric bypass surgery that revealed an inverse relationship between dietary TAC and the amount of PRF in their diet^(35)^. Following gastric bypass surgery, a significant inverse relationship was observed between dietary TAC and the amount of UPF in their diet^(35)^.

Dietary antioxidants consist of vitamins and minerals and a wide range of phytochemicals (e.g. flavonoids, carotenoids and polyphenols)^(36,37)^. Numerous processing techniques can lower the mean TAC for a food item. Specifically, destroying the innate food matrix can decrease antioxidant content^(37,38)^. While some cooking methods are considered minimally processed (e.g. boiling and roasting)^(19)^, cooking is a common processing technique for MPF, which have a higher mean TAC compared with PRF. One possible mechanism explaining the decrease in TAC from MPF to PRF is through thermal processing, which has been shown to decrease the water-soluble bioactive compounds present in MPF^(39)^. For example, cooking, baking and boiling vegetables decrease levels of vitamin C, phenolic compounds and lycopene^(39)^. Specifically through boiling, such antioxidant water-soluble vitamins are lost to the boiling water^(40)^. The effects of temperature-related food processing techniques on TAC are inconsistent across food groups as TAC increases with roasting in specific types of nuts and grains while decreasing in others, depending on the individual heat stability of specific antioxidants present within the food^(29,41,42)^. Similarly, roasting has been found to decrease flavonoid content by up to 33 %, and boiling raw vegetables for 15 min can cause as much as an 82 % loss of antioxidants^(28,42)^. Thus, thermal processing could explain differences between MPF and PRF but does not necessarily explain the totality of findings because some cooking methods are allowed in MPF. While some minimal processing techniques can decrease TAC, simply adding processed culinary ingredients (e.g. oil, butter, salt) re-classifies the food as a PRF. However, the addition of these ingredients does not necessarily decrease TAC. For example, the addition of butter and cooking oils (e.g. olive oil)^(43,44)^ could add fat-soluble antioxidants that may increase TAC. However, further cooking of these culinary foods may in turn decrease TAC. Overall, these findings suggest that any food processing, above minimal, lowers TAC significantly for all food items since no difference was found between PRF and UPF.

The results support the well-established notion that plant-based foods have been identified as rich source of antioxidants^(25)^, which could explain why vegetarians have a higher antioxidant status than non-vegetarians^(45,46)^. Food processing was shown to decrease TAC in plant-based foods, which could be due to their initially high TAC as observed in the present analysis. Although processing of mixed foods that contain both plant and animal-based foods did not lower TAC. The top three categories with the highest mean TAC were plant-based (vegetables, fruits and BNS). Of those groups, fruits was the only category where processing did not result in a lower mean TAC, which contradicts prior research^(26)^ and could result from the direct coding of food items instead of using initial groupings from published data (see methods in^(26)^). Both vegetables and BNS followed the same trend seen in plant-based foods overall (i.e. MPF mean TAC was higher than PRF and UPF). While grains have been identified as sources of antioxidants by others^(47,48)^, the current analysis ranked grains as the lowest plant-based food category and food processing did not alter mean TAC. For beverages, there was no difference between the mean TAC of MPF and PRF, although the mean TAC for UPF was lower. This could be due to the rich antioxidant status of freshly brewed teas and coffee^(49,50)^, with or without added ingredients that increase the overall processing level to PRF. This is another example of how the addition of culinary ingredients, or other MPF, does not necessarily affect the TAC of an overall food (e.g. adding sugar to tea does not change the overall antioxidant content of a food). Since food processing resulted in different mean TAC of some plant-based foods and not others, these results suggest that some processed plant-based food categories can be consumed without altering the overall TAC of a diet (e.g. fruits and grains). However, given the strong evidence linking UPF consumption with poor health outcomes, minimally processed plant-based foods, rich in antioxidants, should primarily be consumed to best promote health.

A major strength of this study was the large database of 1946 food items widely consumed throughout the world. In addition, the high level of initial coding agreement between research pairs provided confidence in the coding outcomes. However, some of the food categories had smaller sample sizes for the different NOVA processing groups (e.g. seven categories for the food processing and food groups analysis had less than twenty-five items). Future research could expand these to better determine the effect of food processing within these food categories. In addition, since some cooking processes are included in MPF, the present results could be limited due to the lack of cooked MPF, without added processed culinary ingredients (e.g. salt, oil and butter), included in the database. The inclusion of mixed food items in this analysis (e.g. mixed foods and mixed food entrees) limits the ability to determine the effect of food processing on these items; however, the majority of food items in the database were not mixed items.

The results of this research provide supporting evidence that diets consisting of mostly processed and UPF have a lower TAC than diets consisting of predominantly MPF processed foods. Since dietary intake of antioxidants and high antioxidant serum levels^(23,51,52)^ may protect against disease and mortality, consumption of minimally processed plant-based foods could promote health and prevent disease. It is currently known that UPF are more energy dense and nutrient poor^(12)^ and provide a significant source of sugar in the diet^(53–55)^, although recent analyses show that the glycaemic index and load of UPF and PRF are lower than MPF^(56)^. In addition to these food characteristics, the lower mean TAC of UPF could be a mechanism through which consumption of these foods promotes inflammatory chronic disease. Decreased consumption of UPF and PRF foods along with increased consumption of MPF foods could increase the antioxidant content of a diet to better promote health and prevent disease. Generally, dietary guidelines promoting the consumption of whole foods already, naturally, push toward eating more antioxidant-rich foods and moving away from processed foods, particularly UPF. The food industry, or potential public health measure and legislation, should consider ways to maintain the original antioxidant content of foods or enrich processed and UPF with antioxidants to increase the healthfulness of foods, without making them too concentrated to avoid negative consequences (see^(57)^). In conclusion, across all foods, and specifically within plant-based foods, any level of processing above minimal decreased mean TAC. However, this trend does not hold for all food categories that could be attributed to the varying food processing techniques or mixtures of foods.

## Supporting information

Basile et al. supplementary material Basile et al. supplementary material

## Supplementary material

For supplementary material/s referred to in this article, please visit https://doi.org/10.1017/S0007114524002800
