# Supplementary figures and images for: Minimally processed foods have a higher total antioxidant content compared to processed and ultra-processed foods: results from an analysis of 1946 food items
Source: Br J Nutr. 2024 Nov 11;132(12):1555–61. doi: 10.1017/S0007114524002800 (PMC11695109; doi:10.1017/S0007114524002800)

Supplemental Figure 1: Data Selection Flowchart


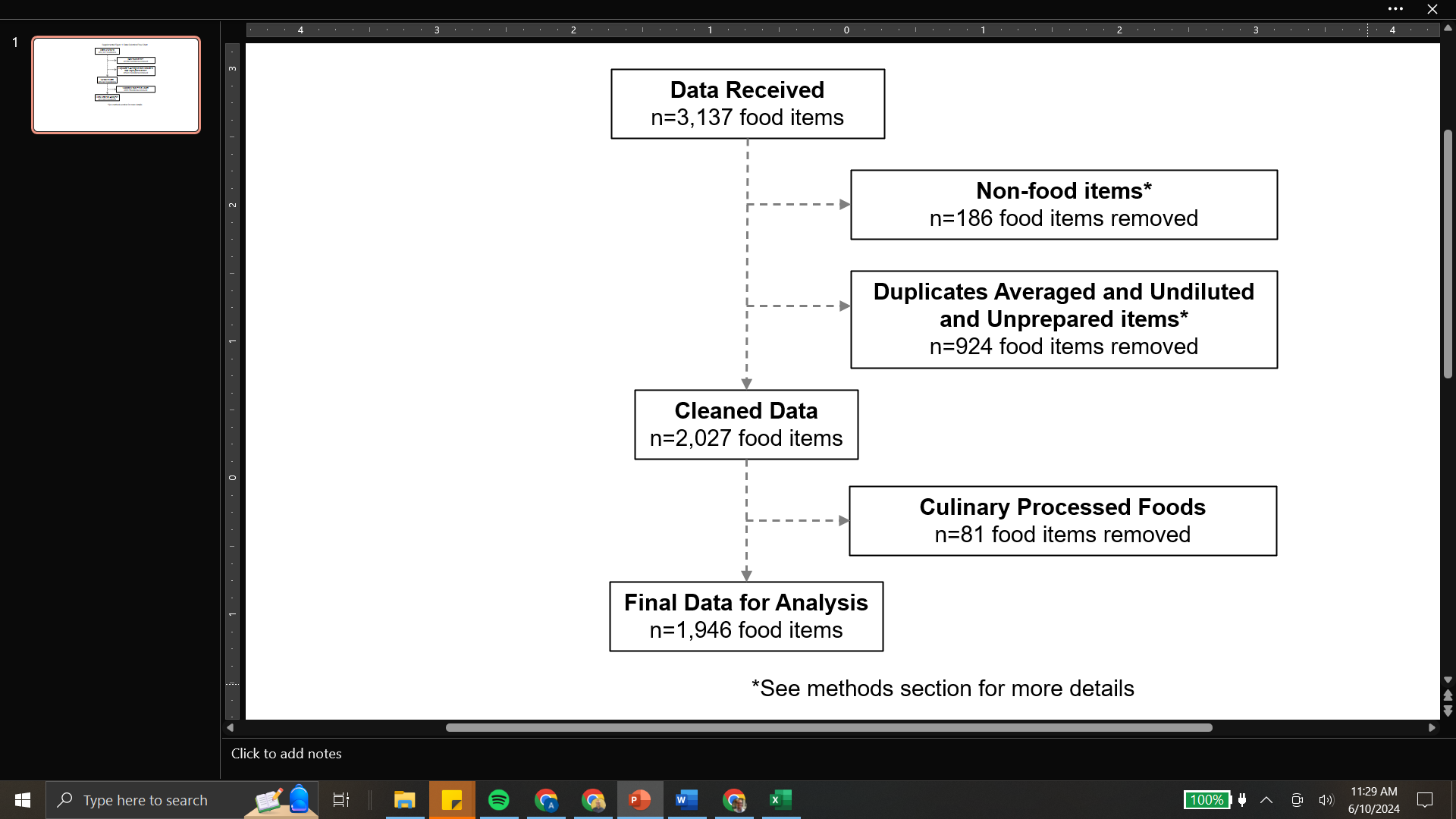

Supplement: Basile et al. supplementary material [file S0007114524002800sup001.docx]
